# Effect of Copper(II) Ion Binding by Porin P1 Precursor Fragments from *Fusobacterium nucleatum* on DNA Degradation

**DOI:** 10.3390/ijms222212541

**Published:** 2021-11-21

**Authors:** Kamila Stokowa-Sołtys, Kamil Wojtkowiak, Valentyn Dzyhovskyi, Robert Wieczorek

**Affiliations:** Faculty of Chemistry, University of Wroclaw, F. Joliot-Curie 14, 50-383 Wroclaw, Poland; kamil.wojtkowiak@chem.uni.wroc.pl (K.W.); valentyn.dzyhovskyi@chem.uni.wroc.pl (V.D.); robert.wieczorek@chem.uni.wroc.pl (R.W.)

**Keywords:** porin protein P1, *Fusobacterium nucleatum*, copper(II) binding, DNA degradation, reactive oxygen species generation, NDMA decomposition

## Abstract

*Fusobacterium nucleatum* is one of the most notorious species involved in colorectal cancer. It was reported that numerous outer membrane proteins (OMP) are actively involved in carcinogenesis. In this paper, the structure and stability of certain complexes, as well as DNA cleavage and ROS generation by fragments of OMP, were investigated using experimental and theoretical methods. Mass spectrometry, potentiometry, UV-Vis, CD, EPR, gel electrophoresis and calculations at the density functional theory (DFT) level were applied. Two consecutive model peptides, Ac-AKGHEHQLE-NH_2_ and Ac-FGEHEHGRD-NH_2_, were studied. Both of these were rendered to form a variety of thermodynamically stable complexes with copper(II) ions. All of the complexes were stabilized, mainly due to interactions of metal with nitrogen and oxygen donor atoms, as well as rich hydrogen bond networks. It was also concluded that these complexes in the presence of hydrogen peroxide or ascorbic acid can effectively produce hydroxyl radicals and have an ability to cleave the DNA strands. Surprisingly, the second studied ligand at the micromolar concentration range causes overall DNA degradation.

## 1. Introduction

Colorectal cancer (CRC) is the third most common cancer [1]. Despite the progress made in terms of diagnosis and treatment methods, it remains one of the leading causes of death among oncological patients (second place worldwide). Among the risk factors, unhealthy diet, obesity, lack of physical activity, postmenopausal hormones, tobacco and alcohol are distinguished [2]. Moreover, gut microbiota play an essential role in the carcinogenesis of the large intestine [3]. Correlations between the composition of human microbiota and CRC were first announced in the 1970s. It was later reported that the existence of more than a dozen bacterial species are associated with a higher risk of colon cancer [4,5]. In 2013, it was concluded that the *Fusobacterium nucleatum* increases neoplastic changes [6,7]. This anaerobic, Gram-negative bacterium is naturally present in human dental plaque. However, if it is present in the colon, it becomes a precursor to cancer. Interestingly, this bacterium is actively involved in cancer progression [8].

It has been reported that numerous *F. nucleatum* outer membrane proteins take part in cancerogenesis [9,10,11,12,13,14]. Moreover, it was shown that fragments of FomA (*F. nucleatum* major outer membrane protein), in the presence of endogenous substances such as hydrogen peroxide or antioxidants, e.g., ascorbic acid, induce cells to generate reactive oxygen species (ROS), leading to oxidative stress. The effect is even more significant in the presence of Cu(II) ions, which form complexes with FomA [13]. The arising ROS can induce DNA damage and trigger redox-dependent transcription factors. The precise mechanism that induces oxidative stress is not fully understood. In addition to causing DNA damage, ROS may further disrupt multiple intracellular signaling pathways. Therefore, the ROS-mediated carcinogenic process is a very complex one [15].

Copper is one of the essential trace metals and is vital to the health of all living organisms. It affects numerous biochemical pathways, not only in the human body [16]. Its deficiency negatively affects the cardiovascular system and bone function [17]. Moreover, it causes weakness, fatigue, skin sores, poor thyroid function and low body temperature [18]. On the other hand, its excess accompanies the development of multiple neoplastic processes, such as intestinal, lung, breast, prostate and brain cancers. Moreover, it causes damage to various tissues and organs [17].

In striking contrast to iron, zinc and manganese, the copper requirements are quite low in most bacteria. According to the Irving-Williams stability series, cupric ions are some of the most stable divalent transition metal ions and possess the highest affinity for metalloproteins [19]. In contrast to eukaryotic cells, most bacteria have a low need to import copper into the cytoplasm. Prokaryotic cells possess all copper-containing enzymes in the cytoplasmic membrane or the periplasmic space [20]. However, the intracellular copper concentrations in numerous bacteria species are two orders of magnitude higher than the extracellular concentrations. As such, copper metalloproteins are used as catalyzers in electron transfer reactions [21], similarly to eukaryotic cells [20]. Despite their importance, it is still unclear how copper ions pass through the bacterial cell membrane [22], although they are probably involved in the Zn(II) uptake system [23]. As with iron and manganese, copper is also potentially toxic because of its ability to generate reactive oxygen species [20]. Due to the limited number of metal ions in the environment and the cytoplasm, it is supposed that proteins compete with other proteins for a limited pool of metal ions rather than metal ions competing with other metals for proteins. Under these conditions, metal occupancy is determined by the relative metal affinities of the different proteins [24]. Therefore, we provide insights into the structure and thermodynamics of Cu(II) complexes with two chosen binding regions from the outer membrane protein P1 precursor from *F. nucleatum*. This protein possesses six histidine residues [25,26], which are often involved in metal binding. However, only four of them are localized in water-soluble regions, namely Ac-AKGHEHQLE-NH_2_ and Ac-FGEHEHGRD-NH_2_. Therefore, most probably they are solvent-exposed and may participate in chelating metal ions. The remaining two histidine residues (Ac-FNHQAKM-NH_2_ and AC-SVAHFIY-NH_2_) are in the hydrophobic fragments of the protein and their accessibility to metal ions is limited. Most likely they are anchored in the cell membrane. Peptides might increase the pro-oxidative activity of metal ions; therefore, the chelation of copper(II) ions can increase the ability to produce reactive species. ROS are indeed a relevant class of carcinogens as powerful oxidizing agents capable of damaging DNA and other important biomolecules. However, in our studies, it was shown that not only does metal ion binding lead to an increased ability for ROS production and DNA relaxation, but also one of the studied ligand itself may cleave plasmid DNA. Therefore, this peptide should be considered a DNA-cleaving agent that may be actively involved in cancer progression. However, this requires further studies. Herein, only general behavior is discussed.

## 2. Results and Discussion

### 2.1. Coordination of Cu(II) Ions at Various pH

Structural and thermodynamical studies of Cu(II) complexes were performed with two model peptides, Ac-AKGHEHQLE-NH_2_ (L^1^) and Ac-FGEHEHGRD-NH_2_ (L^2^), deriving from the outer membrane protein P1 precursor from *F. nucleatum* subsp. *nucleatum*. A broad spectrum of experimental and theoretical methods was applied. The stoichiometry of the formed complexes was assessed based on the mass spectra and potentiometric data. Moreover, based on potentiometric titration, overall stability constants were calculated. Spectroscopic and computational calculations allowed the coordination sphere determination, indicating donor atoms and the geometry of the formed complex species.

**Figure 1:**
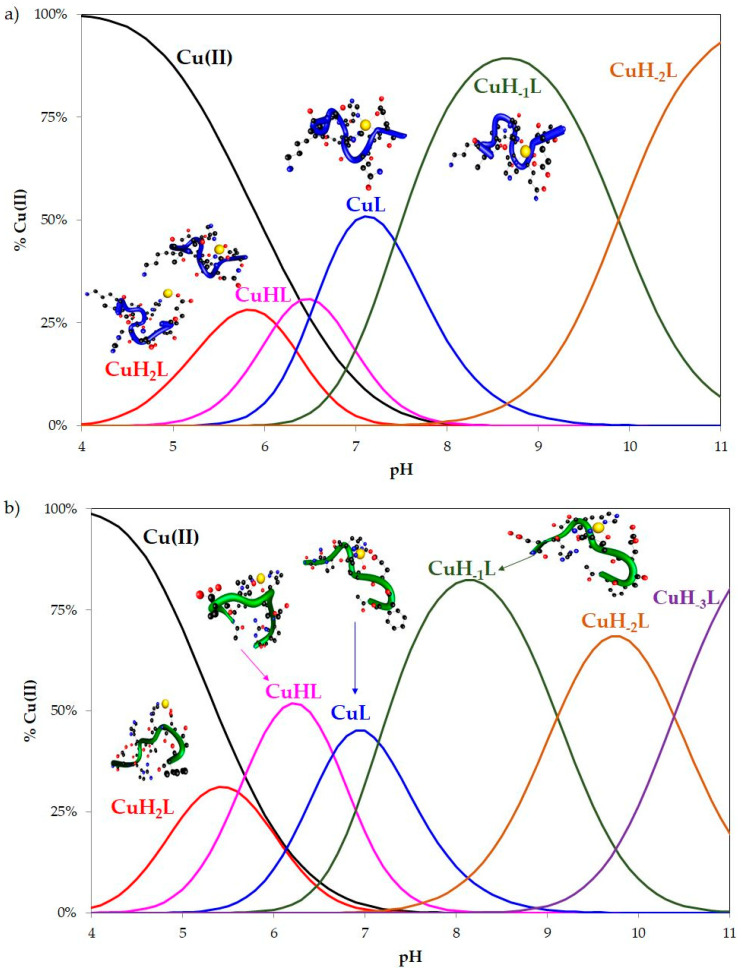
Species distribution diagram for the (**a**) Cu(II)-Ac-AKGHEHQLE-NH_2_ system (L^2^) and (**b**) Cu(II)-Ac-FGEHEHGRD-NH_2_ (L^2^) system. Cu(II):L 1:1.1, [Cu(II)] = 1 mM. Overall stability constants determined at 25 °C and at 0.1 M ionic strength (KCl).

Both investigated peptides form mononuclear complexes with the Cu(II) ion. No bis- or poly-nuclear complexes were detected using either potentiometry (Figure 1) or mass spectrometry.

Titration done with an excess of the metal yielded precipitation of the copper hydroxide. On the other hand, the potentiometric calculations and DFT level of theory did not allow for modeling, which involves more than one ligand in the coordination process. No complexes with 1:2 (CuL_2_) or 1:3 (CuL_3_) metal-to-ligand stoichiometry were detected. Additionally, ESI-MS spectrometry indicated only equimolar complex formation (CuL). No complexes with a higher amount of ligand (CuL_n_, where *n* > 1) were observed. The *m*/*z* values of 545.3 and 562.7 corresponded to [L^1^]^2+^ (monoisotopic mass (M_mi_) equal to 545.3) and [L^2^]^2+^ (M_mi_ = 562.7), respectively. The sodium and potassium adducts of free ligands were also present (*m*/*z* = 556.3 [L^1^ + Na]^2+^ (M_mi_ = 556.3), 564.3 [L^1^ + K]^2+^ (M_mi_ = 564.3), 567.3 [L^1^ + 2Na]^2+^ (M_mi_ = 567.3), and *m*/*z* = 573.7 [L^2^ + Na]^2+^ (M_mi_ = 573.7), 584.7 [L^2^ + 2Na]^2+^ (M_mi_ = 584.7)). The signals deriving only from mononuclear Cu(II) complexes with one attached ligand molecule were located at *m*/*z* values of 575.7 [CuL^1^]^2+^ (M_mi_ = 575.7) and 593.2 [CuL^2^]^2+^ (M_mi_ = 593.2). In the studied systems, the coordination process starts at around pH 4 with CuH_2_L species formation (Figure 1). The charges of the complex species are omitted in this manuscript for the sake of simplicity. In the case of L^1^, deprotonation of the three functional groups within the ligand molecule was observed. However, most probably only two donor atoms are involved in the coordination. According to the equation λ_max_ = 10^3^/[1.18 + 0.052(COO^−^) + 0.140(Im) + 0.166(NH_2_) + 0.200(N=)] the d-d transition at 700 nm observed in UV-Vis spectra (Table 1) suggests the coordination of the oxygen and nitrogen donor atoms in the equatorial plane of the copper(II) ion [27]. The coordination mode was also confirmed by the hyperfine coupling constant equal to 143 Gs and g-factor of 2.335. According to the Peisach-Blumberg plot, the EPR spectra parameters (A_‖_ and g_‖_) corresponded well to metal binding with one nitrogen and oxygen donor atom [28]. Moreover, in the UV-Vis and CD spectra, two charge transfer (CT) bands appeared at around 231 nm and 255 nm, suggesting coordination of the carboxyl group from aspartic acid and N_imidazole_ from the histidine residue, respectively [29,30]. To confirm the coordination pattern, computational methods of theoretical chemistry were used as useful tools to predict the structure and stability of the complexes.

**Figure 2:**
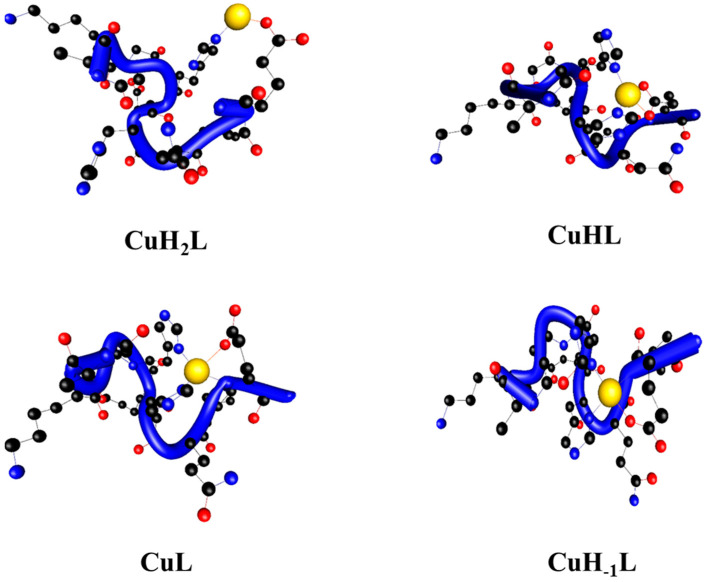
The structures of Ac-AKGHEHQLE-NH_2_ complexes with Cu(II), where blue tubes follow backbones.

**Figure 3:**
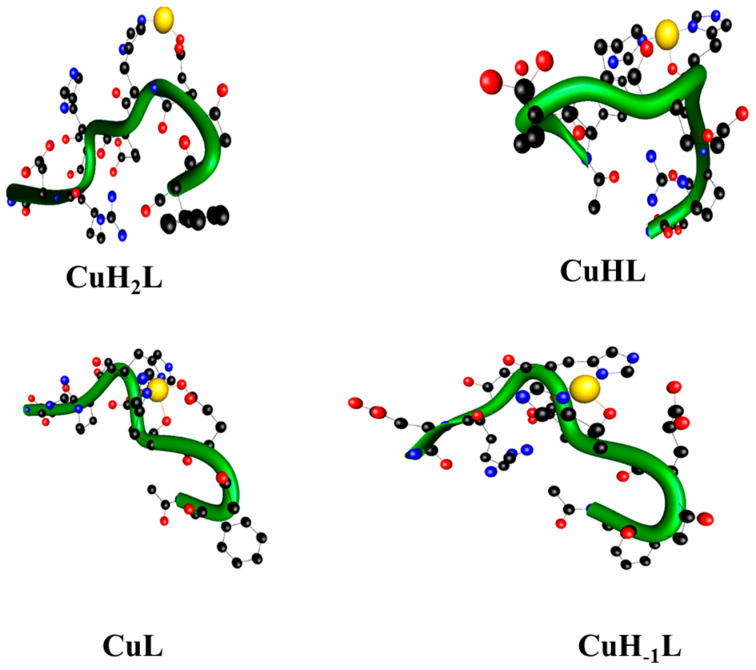
The structures of Ac-FGEHEHGRD-NH_2_ complexes with Cu(II), where green tubes follow backbones.

At the DFT level, multiple connected cation-peptide complexes for both peptides were found (Figure 2 and Figure 3). Experimental data combined with density functional theory (DFT) allowed for the histidine residue responsible for metal ion coordination to be determined. Theoretical calculations revealed that in the CuH_2_L^1^ complex, the cation binds with the ligand through the imidazole ring of H6, which is supported by one metal oxygen interaction from the side chain of the E9 residue (see Table 2). The remaining coordination sites in the metal ion stay occupied by water molecules to be consistent with the coordination number of the Cu(II) ion.

For the second studied ligand L^2^, four protons are lost in the first complex form, namely three carboxylic groups and an imidazole ring are deprotonated. The CuH_2_L^2^ complex binds the cation using the imidazole ring of the H4 with one supporting interaction between the oxygen atom of the side chain of E3 and the copper cation (Table 2). These theoretical results were confirmed by spectroscopic parameters of the species. In the UV-Vis spectra, a band at 707 nm with ε equal to 38 M^−1^ cm^−1^ was observed. The presence of a charge transfer band (COO^−^→Cu(II)) at 238 nm in the CD spectra, as well as a shoulder band at 250 nm (N_im_→Cu(II) [27,31], further proved the above presumption (Table 1).

The constants (pKa) accompanying the dissociation of another functional group and formation of the CuHL^1^ and CuHL^2^ species in the general reaction CuH_2_L ↔ CuHL + H^+^ are 6.13 and 5.61, respectively (Table 1). The pKa value of this deprotonation step is much lower than the one detected in the free ligands (6.86 and 6.93, respectively; Table S1) and most likely corresponds to the binding of an imidazole nitrogen to the Cu(II) ion [32]. Therefore, potentiometric data confirm the participation of these moieties in the coordination. Moreover, in the UV-Vis spectra, a hypsochromic shift of d-d band was observed. However, CuHL^1^ coexists in solution with the major CuL^1^ species (which reaches a maximum at pH 7.1), meaning its full spectroscopic characterization was impossible and only EPR parameters could be obtained. An increase in the A_‖_ value to 167 with a simultaneous decrease in g_‖_ to 2.300 suggested {2N_im_, 2O_COO_-} coordination mode [28]. The second studied complex, CuHL^2^, predominates at pH 6, with an abundance of around 52%. Its formation is accompanied by shifting of the d-d band for about 100 nm toward a shorter wavelength (from 707 nm to 610 nm). Moreover, in the CD spectra, a more intense negative Cotton effect could be seen at 250 nm, while no new band appeared. Additionally, the EPR parameters changed, similarly to CuHL^1^. However, A_‖_ and g_‖_ values were a little bit lower for CuHL^2^ than for CuHL^1^, probably because only one carboxylic group took part in metal ion binding. These results suggest that copper(II) binding occurred caused by two nitrogen donor atoms derived from histidine residues and the side-chain carboxylic group of aspartic acid [33,34]. Therefore, computational methods were applied to distinguish the coordination mode. It was concluded at the DFT level that the CuHL^1^ complex binds two nitrogen atoms of the imidazole groups of the H4 and H6 histidines, while two supporting Cu(II).O interactions with both oxygen atoms of the E9′s side chain also occur. Interestingly, the CuHL^1^ complex was the only one we found where two supporting interactions come from the side chain of E9. In the CuHL^2^ complex, the second imidazole ring is involved and both H4 and H6 histidines stabilize the complex. Only one oxygen-metal-supporting interaction was found here—the carbonyl oxygen of H6 with a bond length of 1.834 Å.

In both peptides, apart from the two available imidazole nitrogen (from histidine residues) and carboxylic groups (from aspartic acid residues), the Cu(II) ion can also be bound to the amides of the peptide bonds located in the direction of the N- or C-terminus. At pH values above 6.5, CuHL^1^ and CuHL^2^ complexes lose the proton and the CuL^1^ and CuL^2^ species are formed, respectively. The d-d transition energy values at 600 nm for CuL^1^ and 575 for CuL^2^ in UV-Vis spectra and the presence in CD spectra of charge transfer transitions of COO^-^Cu(II) at around 220 nm for CuL^1^, 235 nm for CuL^2^, N_am_^−^→Cu(II) at around 330 nm (which overlaps with N_im π1_→Cu(II) transition) and N_im π2_→Cu(II) at around 250 nm, as well as EPR parameters A_‖_ = 185 Gs and g_‖_ = 2.264 for CuL^1^ and A_‖_ = 180 Gs, g_‖_ = 2.215 for CuL^2^, correspond very well to the {2N_im_, N_am_^−^, O_COO_-} coordination mode [35]. The CuL^1^ is formed similarly to the CuHL^1^: two imidazole rings and amide nitrogen from the E9 residue are supported by one metal-oxygen interaction. The different folding modes of the L^1^ and L^2^ ligands make it possible to bind different amide nitrogen atoms. The CuL^2^ complex is created by both the imidazole rings, H6 carbonyl oxygen and H4 amide nitrogen atom. Please note that in contrast to the CuL^1^ complex, the 3N set of interactions in CuL^2^ is supported by two Cu(II).O bonds, namely the H6 carbonyl and E3 carboxylate groups.

The next proton is released from another amide bond (pKa = 7.43 and 7.15 for CuH_-1_L^1^ and CuH_-1_L^2^ formation, respectively) resulting in the {2N_im_, 2N_am_^−^, O_COO_-} coordination mode. The second amide binding to copper(II) ion is supported by the enhanced positive Cotton effect at around 290 nm in CD spectra of both complexes. A significant change in the geometry of the formed complexes can also observed. This manifests in Cotton effects in CD spectra. The split of the d-d band is a result of the decreasing symmetry within the forming complex [36,37]. The CD spectra show signs of d-d transitions typical for Cu(II) square planar complexes [38]. The EPR spectra parameters also imply the engagement of four nitrogen donor atoms and one oxygen in the coordination process [39]. In order to precisely define which donor atoms are involved in the coordination, the theoretical methods were used. In the CuH_-1_L^1^ complex, a set of five interactions contains two imidazole-metal interactions, two amide nitrogen-metal interactions from L8 and E9 residues and one metal-oxygen interaction from the side chain of E9. Please note that interactions with E9 and H6 residues are present in all investigated complexes. The O1 atom from E9 supports all complexes. The interaction in the amide nitrogen of E9 is also present in CuHL^1^ and CuH_-1_L^1^ complexes for H6 residue, whereby the imidazole ring binds cation in all complexes. The CuH_-1_L^2^ complex acting to bind metal cations engages in the same set of ligand atoms, with CuL^2^ plus one additional amide nitrogen from H6. The most common type of interaction in the series of investigated complexes is the interaction between the metal cation and nitrogen atom of the imidazole ring. Such interactions are supported by metal-oxygen bonds in all investigated complexes. The rich interaction pattern follows 2,4,4,5 and 2,3,5,5 interaction numbers for L^1^ and L^2^ complexes, respectively. Due to the different atom sets in the investigated complexes, a direct comparison of the energy is not possible. However, using the type, the number and bond lengths were used to estimate the relative stability of the complexes as follows: CuH_2_L < CuHL < CuL < CuH_-1_L.

It is worth noting that the spectroscopic parameters do not change significantly when raising the pH value of the solution. The deprotonation of CuH_-1_L to CuH_-2_L (pKa = 9.88 and 9.11 for CuH_-1_L^1^ and CuH_-1_L^2^, respectively) correspond to the removal of a proton from the amino group or the guanidinium moiety of basic amino acid residues from L^1^ and L^2^, respectively. The lower pKa value in this process compared to the metal-free ligand L^2^ (9.63, Table S1) may suggest some involvement of the Arg side chain in the interaction with the metal ion [40]. Moreover, diminution of the pKa value may be a result of burying the Arg residue inside the protein. It is evidenced that such basic groups have much lower pKa values in comparison to free amino acids [41]. A similar situation can be observed for the ligand alone (see Table S1 in the Supplementary Materials). The last step of the dissociation of CuH_-2_L^2^ to CuH_-3_L^2^ is observed only for the second studied complex. The process is characterized by pKa values equal to 10.39. This additional deprotonation constant for the complex is higher than the last one for free ligands (highest value = 9.63; Table S1). Therefore, the stepwise deprotonation constant most likely corresponds to a water molecule bound apically to the complex core [42]. This hypothesis is supported by the absence of spectroscopic changes during the formation of the CuH_-3_L^2^ complex, thereby excluding the coordination of an additional donor atom. The consistency of the spectroscopic parameters implies no changes in the coordination mode (Table 1) [38].

**Figure 4:**
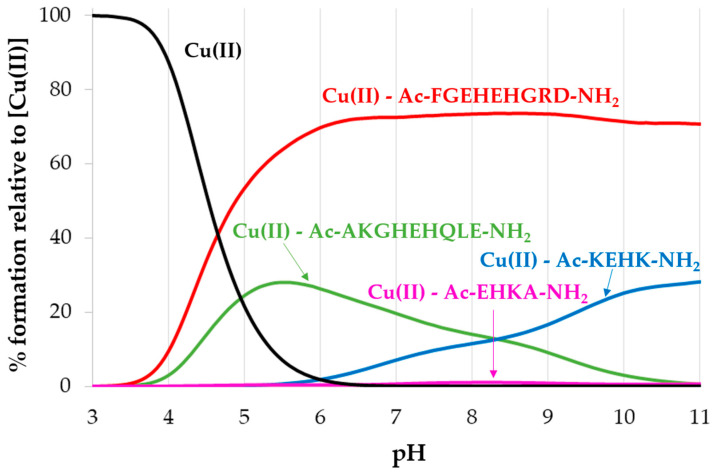
The competition plot for Cu(II), Ac-AKGHEHQLE-NH_2_, Ac-FGEHEHGRD-NH_2_, Ac-KEHK-NH_2_ and Ac-EHKA-NH_2_ with M/L molar ratio of 1:1, [Cu(II)] = 1 mM.

The diagram depicted in Figure 4 shows the competition for metal binding among peptides derived from porin P1 and its precursor. The data for Ac-KEHK-NH_2_ and Ac-EKHA-NH_2_ were already published [43] and are used herein only to prepare the competitive diagram of copper(II) speciation among peptides: Ac-AKGHEHQLE-NH_2_, Ac-FGEHEHGRD-NH_2_, Ac-KEHK-NH_2_ and Ac-EHKA-NH_2_. The most effective metal ion binding was observed for the peptide Ac-FGEHEHGRD-NH_2_, in which complex species were stabilized by a rich hydrogen bond (HB) network (Table 3 and Table 4).

The intramolecular HBs can provide significant additional stability to the peptide complexes. We observed here only complexes with one type of hydrogen bond (O..H-N). However, the origins of the proton donor and proton acceptor differ in most cases. The proton acceptor can be provided by the ligand backbone as well as side chains. Commonly, the oxygen atom from the carbonyl group plays the role of the proton acceptor. The intramolecular O H-N HBs of the backbone can stabilize ligands at ~5 kcal/mol per HB. One shall expect that this interaction to provide helical fragments of the ligand. We found hydrogen bonds in all Cu(II)-L^1^ complexes (Table 3). Both CuH_2_L^1^ and CuHL^1^ complexes are stabilized by a set of four hydrogen bonds. In the CuL^1^ and CuH_-1_L^1^ complexes, only two hydrogen bonds were found. As expected, we observed a decreasing number of HBs, since the short ligand L^1^ builds multiple metal-ligand interactions in CuH_2_L^1^ and CuHL^1^ complexes that make the backbone more rigid. Interestingly, we found alpha helical fragments in almost all complexes (CuH_-1_L^1^ was the exception here). Please note that in the CuH_2_L^1^ and CuL^1^ complexes, only one alpha-helical-type hydrogen bond exists, however in CuHL^1^ the shortest possible (two members) cooperative chain of hydrogen bonds is created (K2..E5..L8).

In comparison to the Cu(II)-L^1^ complexes, the Cu(II)-L^2^ complexes form a significantly richer HB network (see Table 4). The number of stabilizing hydrogen bonds for the whole series is seven (for CuH_2_L^2^ and CuH_-1_L^2^) or six (for CuHL^2^ and CuL^2^). This HB stabilization is possible due to the presence of arginine, which is responsible for the building of 50% or more hydrogen bonds in each complex. In two complexes (CuH_-1_L^2^ and CuHL^2^) we found short helical fragments. The Cu(II)-L^2^ complex builds two 3-10 helix-type hydrogen bonds; however, in contrast to Cu(II)-L^1^, they are separated and do not form a cooperative chain. CuH_-1_L^2^ contains one alpha-helical-type hydrogen bond with a typical length of ~1.8 Å.

For both ligands we spotted only one type hydrogen bond—O..H-N—with great contributions from proton donors and proton acceptors form side chains of the ligands. We expect that the stabilization originating from the hydrogen bond network will be greater for complexes with L^2^ ligands due to the greater number of HB interactions.

### 2.2. Oxidative Properties

**Figure 5:**
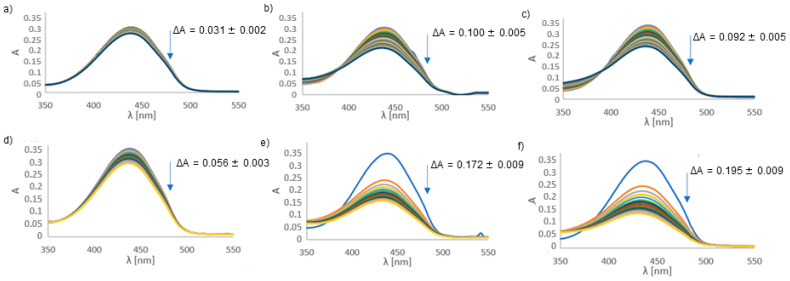
UV-Vis spectra of NDMA in the presence of hydrogen peroxide: measured for one hour every 1 min with (**a**) copper (II) ions (control), (**b**) Cu(II)-L^1^ complex and (**c**) Cu(II)-L^2^ complex; measured every hour for 15 h with (**d**) copper (II) ions (control), (**e**) Cu(II)-L^1^ complex and (**f**) Cu(II)-L^2^ complex.

The UV-Vis spectra of the reporting molecule, NDMA (N,N-Dimethyl-4-nitrosoaniline), in the presence of hydrogen peroxide and the studied complexes are shown in Figure 5. The spectra were measured to detect the presence of hydroxyl radicals. Comparing the differences in absorbance between the control system (Figure 5a) and the systems containing the Cu(II)-L^1^ (Figure 5b) and Cu(II)-L^2^ (Figure 5c) complexes, it can be concluded that the decrease in absorbance was more than three times greater for systems containing complexes than for free cupric ions. This proves that complex formation promotes reactions during which hydroxyl radicals are generated. The same effect was observed when the reaction time was extended to 15 h. The difference in absorbance was again three times greater than for the control, meaning in the system with the analyzed complexes, a greater amount of the hydroxyl radical was formed. It can be noticed that most radicals were formed in the first hour, then with each subsequent hour the reaction became slower and slower, as evidenced by the lower decrease in absorbance. The results suggest that complexes in the presence of hydrogen peroxide form a significant amount of hydroxyl radical, the most reactive ROS, making the environment more pro-inflammatory [12].

**Figure 6:**
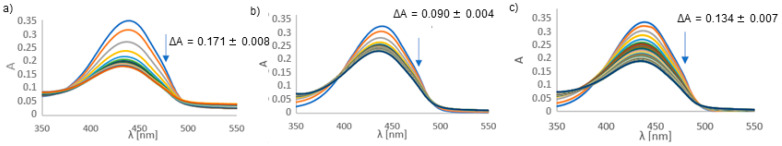
UV-Vis spectra of NDMA in the presence of ascorbic acid measured for one hour every 1 min with (**a**) copper (II) ions (control), (**b**) Cu(II)-L^1^ complex and (**c**) Cu(II)-L^2^ complex.

Interestingly, in the presence of ascorbic acid (Figure 6), the copper complexes act as antioxidants, preventing the formation of the hydroxyl radical. A significant amount of hydroxyl radical is formed during the control reaction, in which copper ions are reduced by ascorbate. However, when copper ions are bound in the complex, the reduction reaction is not as efficient. This results in the formation of a smaller amount of the hydroxyl radical. At pH 6.8 (the physiological pH value of the colon), at which the tests were carried out, the CuL complex dominates in the solution. In this species, the carboxyl group is bound to the copper ion. Therefore, this negatively charged group can repel ascorbate. Moreover, there is a positively charged arginine residue on the other side of the CuL^2^ complex, away from the copper(II) ion. This group can attract the ascorbic anion and form an ionic bond with it. Therefore, Asc cannot reduce the copper(II) ion, and consequently the Fenton reaction does not occur, hydroxyl radicals are not generated and bleaching of NDMA is not observed.

**Figure 7:**
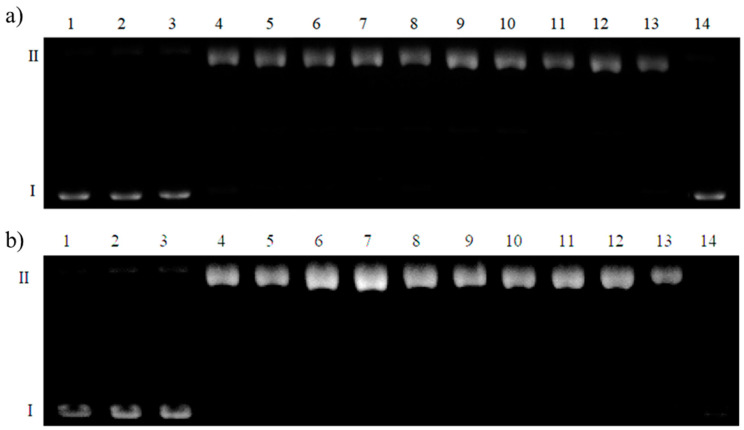
Degradation of plasmid DNA in the presence of copper(II) complex with (**a**) Ac-AKGHEHQLE-NH_2_ (L^1^) and (**b**) Ac-FGEHEHGRD-NH_2_ (L^2^) in the presence of 50 µM H_2_O_2_. The samples were incubated for one hour at 37 °C. Lane 1: plasmid; lane 2: plasmid + 10 µM Cu(II) + 50 µM H_2_O_2_; lane 3: plasmid + 10 µM complex + 50 µM H_2_O_2_; lane 4: plasmid + 20 µM Cu(II) + 50 µM H_2_O_2_; lane 5: plasmid + 20 µM complex + 50 µM H_2_O_2_; lane 6: plasmid + 50 µM Cu(II) + 50 µM H_2_O_2_; lane 7: plasmid + 50 µM complex + 50 µM H_2_O_2_; lane 8: plasmid + 100 µM Cu(II) + 50 µM H_2_O_2_; lane 9: plasmid + 100 µM complex + 50 µM H_2_O_2_; lane 10: plasmid + 500 µM Cu(II) + 50 µM H_2_O_2_; lane 11: plasmid + 500 µM complex + 50 µM H_2_O_2_; lane 12: plasmid + 1000 µM Cu(II) + 50µM H_2_O_2_; lane 13: plasmid + 1000 µM complex + 50 µM H_2_O_2_; lane 14: plasmid + 1000 µM ligand + 50 µM H_2_O_2_.

The effects of the complexes and free copper(II) ions at the corresponding concentrations were assessed by performing gel electrophoresis (results shown in Figure 7). The first path was a control reaction, while on the remaining odd paths, systems containing the complex were separated and the even lanes contained free copper(II) ions. The last lane contained a ligand with no metal ions added. The lowest concentration (10 µM) of both copper ions and the complex had no impact on the DNA structure. Only the circular form of DNA remained in the following paths (lanes 4–9), at similar amounts for the even and odd paths. It can be assumed that free copper(II) ions and complex in a concentration range of 20–100 µM show similar effects on DNA. By comparing lanes 10 with 11 and 12 with 13, it can be seen that in the odd lanes, bands derived from form II are less bright. Therefore, the complex degraded DNA molecules into short polynucleotide fragments. Thus, the effect on DNA relaxation and degradation was more pronounced for complexes than free cupric ions.

Surprisingly, as shown in Figure 7b (lane 14), the ligand itself completely degraded the DNA. The same concentration of the complex and free peptide damaged DNA to different extents. Stronger cleavage of the plasmid by the ligand indicates that (i) the sample is contaminated with protein DNases or (ii) the DNA cleavage mechanism involves non-coordinating side chains of amino acid residues. When functional groups are involved in the coordination process, their free electron pairs are engaged in coordination bond formation and cannot participate in the DNA degradation mechanism.

**Figure 8:**
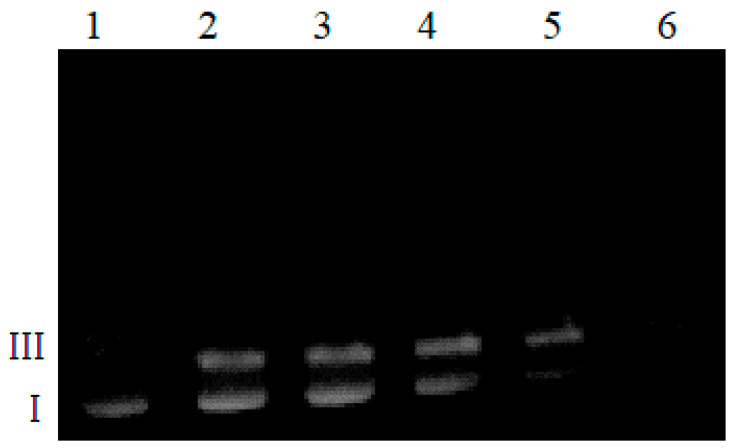
Plasmid DNA cleavage in the presence of Ac-FGEHEHGRD-NH_2_ (samples incubated for one hour at 37 °C). Lane 1: plasmid; lane 2: plasmid + 10 µM ligand; lane 3: plasmid + 50 µM ligand; lane 4: plasmid + 100 µM ligand; lane 5: plasmid + 500 µM ligand; lane 6: plasmid + 500 µM ligand.

The data from the previous experiment prompted us to investigate the activity of L^2^ alone more thoroughly. The results depicted in Figure 8 show the substantial extent of plasmid degradation observed at the 10 µM concentration for the L^2^ ligand (lane 2). The damage was double-stranded and the cleavage mechanism was not gradual; hence, no bands indicating the presence of form II are visible. Cuts of both strands at the interaction site were detected. The degree of DNA damage depends on the ligand concentration. In the presence of 500 µM L^2^, total degradation of DNA can be observed. It can also be concluded that the genetic material damage is not associated with oxidizing conditions (a similar experiment in the presence of 50 μM H_2_O_2_ was performed). Regardless, this is an unusual feature of the studied ligand. Additionally, pre-incubation of the studied ligand solution under conditions where a majority of the protein DNases undergo deactivation (5 min, 100 °C) [44] did not influence its ability to destroy DNA. The positively charged arginine residue most probably interacts with negatively charged DNA, and the DNA cleavage reaction proceeds via acid-base catalysis in which the imidazole ring is involved. Imidazole is an efficient catalyst of ester hydrolysis at neutral pH, which catalyzes the hydrolysis of RNA and its various derivatives [45]. While many reagents have been successfully applied for the hydrolytic cleavage of RNA, there have been fewer successes with DNA, because of its relatively high hydrolytic stability [46,47]. However, histidine residues are often involved in natural hydrolytic metalloenzyme active centers (e.g., ribonuclease). Moreover, histidine-containing peptides have potential to bind specifically to DNA and hydrolyze the DNA-phosphodiester bond [48,49].

**Figure 9:**
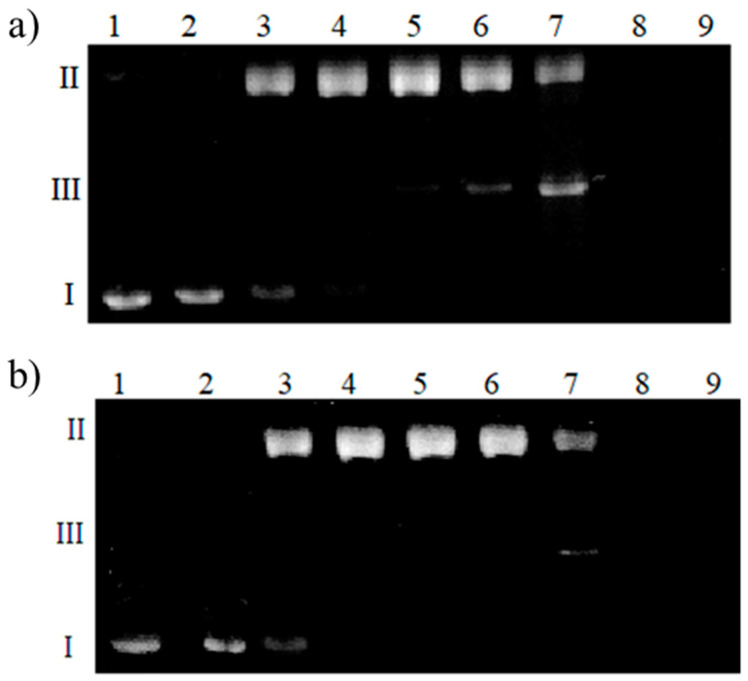
Plasmid DNA degradation in the presence of (**a**) Cu(II)-L^1^ and (**b**) Cu(II)-L^2^ complexes with different concentrations of ascorbic acid. Lane 1: plasmid; lane 2: plasmid + 50 µM complex; lane 3: plasmid + 50 µM complex + 5 µM Asc; lane 4: plasmid + 50 µM complex + 10 µM Asc; lane 5: plasmid + 50 µM complex + 25 µM Asc; lane 6: plasmid + 50 µM complex + 50 µM Asc; lane 7: plasmid + 50 µM complex + 100 µM Asc; lane 8: plasmid + 50 µM complex + 250 µM Asc; lane 9: plasmid + 50 µM complex + 500 µM Asc.

The electropherogram depicted in Figure 9 shows the effects of complexes in the presence of ascorbic acid on the plasmid DNA structure.

The first lane shows DNA control, while the second one reveals the impact of the complex itself. Lanes 3 to 9 show the influence of the complexes in the presence of increasing Asc concentration. The lowest concentration of ascorbic acid causes single-stranded cuts of DNA, which are visible as the bright shining form (II). The 10–25 µM concentration of ascorbic acid caused the complete conversion of the super-helical form of DNA to the circular one. However, a small amount of linear form III can be seen in lane 6 for Cu(II)-L^1^ (Figure 9a). Therefore, 50 µM ascorbic acid induced double-stranded DNA cleavage. For Cu(II)-L^2^, a two-fold greater Asc concentration is needed for a similar effect. Higher Asc concentrations cause total DNA degradation to short polynucleotide fragments; hence, lanes 8 and 9 show no visible bands indicating the presence of any form of DNA. As can be seen from the DFT structure for CuL^1^ and CuL^2^ (Figure 2 and Figure 3, respectively), a negatively charged carboxyl group is bound to the copper(II) ion in both complexes. These negatively charged groups repel the ascorbate from the metal ion. This prevents the reduction of metal ion and ROS are not formed very efficiently. However, comparing DFT structures of Cu(II)-L^1^ to Cu(II)-L^2^, it can be easily seen that in the case of Cu(II)-L^2^, two negatively charged carboxyl groups are close to the copper(II) ion. Moreover, positively charged Arg residue may attract Asc. Therefore, all Cu(II)-L^2^ species exhibit lower DNA damaging properties in the presence of ascorbic acid.

## 3. Materials and Methods

### 3.1. Materials

The studied peptides Ac-AKGHEHQLE-NH_2_ (L^1^) and Ac-FGEHEHGRD-NH_2_ (L^2^) were purchased from KareBay Biochem, Inc. (Monmouth Junction, NJ, USA). The pBR322 plasmid DNA from *E. coli* RRI buffered solution was obtained from Sigma-Aldrich (Saint Louis, MO, USA), while other chemical reagents (e.g., CuCl_2_, NaOH, HCl, H_2_O_2_, Asc) were acquired from commercial sources, mainly from Merck (Darmstadt, Germany).

### 3.2. Mass Spectrometry 

ESI-MS experiments were performed on the LCMS-9030 qTOF Shimadzu (Shimadzu, Kyoto, Japan) device, equipped with a standard ESI source and the Nexera X2 system. Analysis was performed in the positive ion mode between 100 and 3000 *m*/*z*. The LCMS-9030 parameters were: nebulizing gas—nitrogen, nebulizing gas flow—3.0 L/min, drying gas flow—10 L/min, heating gas flow—10 L/min, interface temperature 300 °C, desolvation line temperature—400 °C, detector voltage—2.02 kV, interface voltage 4.0 kV, collision gas-argon, mobile phase 50% A in B (where A is water + 0.1% acetic acid and B is acetonitrile + 0.1% acetic acid), mobile phase total flow—0.3 mL/min. The injection volume was within the range of 0.1 to 1 μL, depending on the intensity of the signals observed on the mass spectrum. All obtained signals had mass accuracy errors in the range of 1 ppm. The concentration of peptide was 1 10^−4^ M with a M/L molar ratio of 1:1. Samples were prepared in the water/acetonitrile (50/50 *v*/*v*) mixture at pH 7. All of the used solvents were of LC-MS grade. The obtained data were analyzed and simulated using an ACD/Spectrus processor (ACD/Labs, Toronto, ON, Canada).

### 3.3. Potentiometric Measurements

Overall stability constants for proton and metal complexes were calculated from titration curves registered over the pH range of 2.5–10.5 at 25 °C, in 5 mM HCl water solution and ionic strength of 0.1 M (NaCl), using a total volume of 1.5 mL. Potentiometric titrations were performed using a Metrohm 905 Titrando (Metrohm AG, Herisau, Switzerland) pH meter system provided with a Mettler-Toledo InLabMicro (Mettler-Tololedo, Columbus, OH, USA) combination pH electrode. High-purity grade argon was gently blown over the studied solution (to maintain an inert atmosphere). Solutions were titrated with 0.1003 M carbonate-free NaOH. The electrode was calibrated daily for hydrogen ion concentration by titrating HCl with an alkaline solution. The ligand concentration was 1.1 mM and the metal-to-ligand ratios were 1:1.1 and 1:3. The standard potential and the slope of the electrode were computed with Glee software. The purities and exact concentrations of the ligand solutions were determined in SUPERQUAD [50] using the Gran method. The HYPERQUAD 2006 [51] program was employed for the overall (β) stability constant calculations. The reported pKa values represent the acid dissociation constants of the corresponding species. The computed standard deviations (referring to random errors only) were given by the program itself and are shown in parentheses as uncertainties in the last significant figure.

### 3.4. Spectroscopic Studies

Electronic absorption (UV-Vis) spectra were recorded using a Cary 60 spectrophotometer (Agilent Technologies, Santa Clara, CA, USA) and circular dichroism (CD) spectra measurements were performed on a Jasco J-1500 spectropolarimeter (JASCO Corporation, Tokyo, Japan), both at 25 °C. The direct CD measurements (θ (mdeg)) were converted to the mean residue molar ellipticity (Δε (M^−1^ cm^−1^)) using a Jasco Spectra Manager (JASCO Corporation, Tokyo, Japan). In both spectroscopic techniques, the spectra were measured in two quartz cuvettes with optical path lengths (OPL) of 1.0 and 0.1 cm in the 300–800 and 200–400 nm ranges for each cuvette, respectively. The ligand concentration was 1.1 × 10^−3^ M and a metal-to-ligand molar ratio of 1:1.1 was used.

The EPR spectra were recorded on a Bruker spectrophotometer (Bruker ELEXSYS E500 CW-EPR, Bruker, Billerica, MA, USA) in the X-band frequency (9.5 GHz). The molar ratios were the same as those used for UV-Vis and CD measurements. However, The concentrations of samples were (Cu(II)) = 3 × 10^−3^ M. Ethylene glycol was added to each sample to obtain homogeneity (3:10, *v*:*v*). The samples were frozen and the measurements were carried out at −19 °C (liquid nitrogen). All EPR parameters were calculated through spectral simulation in the Doubletexact system (SPIN = 1/2: EXACT) as implemented by Andrew Ozarowski, National High Field Magnetic Laboratory, University of Florida, for the spectra obtained at the maximum concentration of the particular species.

Measurements were carried out in the pH 3.0–11.0 range using Mettler-Toledo SevenEasy pH meter (Mettler-Tololedo, Columbus, OH, USA) equipped with a combined glass-Ag/AgCl electrode (InLab Micro, Mettler-Toled, Columbus, OH, USA). The strong acid and base solutions were used in order to change the pH values. All measured spectra were used to determine the coordination processes of cupric ions to the peptides with increasing pH values.

### 3.5. Theoretical Studies

Computational theoretical chemistry methods were used as useful tools to predict the structure and stability of the ligands and complexes [50,51,52,53,54,55,56,57,58]. Molecular orbital studies on 1:1 Cu(II) cation complexes with L^1^ and L^2^ ligands were performed at the DFT level, with the IEFPCM [59] solvent (water) model introduced for potential energy surface investigations. The starting structure of the peptide for DFT calculations was generated on the basis of the amino acid sequence after 75 ps simulation at 300 K, without cutoffs using BIO+ implementation of the CHARMM force field. DFT calculations were performed with the Gaussian 09 C.01 [60] suite of programs using the ωB97X-D [61] long-range-corrected hybrid density functional with damped atom-atom dispersion corrections and a double-ζ 6–31G(d,p) basis set containing polarization functions. All presented structures were and thermodynamically stable.

### 3.6. DNA Cleavage and Reactive Oxygen Species Detection

The ability of Cu(II) complexes of studied peptides to induce single- and double-strand breaks in the absence and presence of H_2_O_2_ or ascorbic acid was tested with the pBR322 plasmid DNA from *E. coli* RRI (Merck). The molar ratios of M/L 1:1 reagents were at the final concentrations described under each electropherogram. The samples were dissolved in 50 mM phosphate buffer at pH 6.8 containing DNA (25 μg/mL). After 1 h incubation at 37 °C, reaction mixtures (20 μL) were mixed with 4 μL of loading buffer (bromophenol blue in 30% glycerol) and loaded on 1% agarose gels containing ethidium bromide in TBE buffer (90 mM Tris/borate, pH 8.0; 20 mM EDTA). Gel electrophoresis was performed at a constant voltage of 4 V cm^−1^ for 180 min. The gels were photographed and processed with a Digital Imaging System (Syngen Biotech, Wroclaw, Poland). Results were obtained in triplicate for all experiments. Moreover, when the same concentration of the reagents was used in several experiments, the view of the lanes was compared with the obtained electropherograms.

### 3.7. ROS Generation Measurements

NDMA, a scavenger molecule used commonly in studies on hydroxyl radicals and reactive species, was used in the spectroscopic absorption experiments. The measurements were followed at 37 °C on a Cary 60 spectrophotometer equipped with a Single-Cell Peltier Accessory (Agilent Technologies) in 1 cm cuvettes at 440 nm, which is a characteristic wavelength of NDMA absorption. The reaction mixtures contained NDMA (25 μM), peptide, Cu(II) (each 50 μM) and H_2_O_2_ or Asc (0.5 mM), all in sodium phosphate buffer (50 mM). Data are reported as the averages of three trials with standard deviations shown in the figures.

## 4. Conclusions

Cancer is now one of the leading causes of morbidity and mortality worldwide, with an estimated 18 million new cases and almost 10 million deaths in 2018. Globally, about 1 in 6 deaths is due to cancer. It is predicted that by 2030, the number of new cancer cases will increase to more than 21 million and cancer-related deaths will increase to 13 million per year [62]. In many cases, human microbiota are associated with cancer progression, e.g., *F. nucleatum* promotes colon cancer growth and even metastasis [63]. Microbial pathogens drive tumorigenesis in 15–20% of cancer cases [64]. On the other hand, reactive species are indeed a relevant class of carcinogens as powerful oxidizing agents capable of damaging DNA and other important biomolecules. Therefore, physiological ROS generation may account for the enlarged risk of cancer development in the aged, while the increased formation of ROS promotes the development of malignancy. It was concluded that outer membrane proteins from *F. nucleatum* stimulated colon cancer cells to produce reactive oxygen species. They also displayed the ability to induce oxidative stress inside these cells [65]. Moreover, we proved that *F. nucleatum* strains in the presence of cupric ions and H_2_O_2_ generate free radicals outside the bacterial cells [66]. The present study shows that ROS production is very efficient when copper(II) ions are bound to outer membrane proteins. As a transition metal ion, copper(II) can promote a Fenton-like reaction that leads to high amounts of reactive oxygen species. If ROS are not quenched by antioxidants, they can damage cells at different sites and in different ways. In the case of DNA, unrepaired damage can lead to the initiation and progression of the carcinogenesis process. It should be stressed that dietary copper is absorbed partially in the stomach. However, the largest portion passes into the duodenum and ileum, which are the major sites of absorption. As copper reaches the duodenum, it comes in contact with copper transporter 1, which is the primary protein responsible for the import of dietary copper across the brush border microvilli. Due to the long retention time of high copper foods (e.g., organ meats, red meat) in the colon (12–30 h) and the presence of endogenous hydrogen peroxide, which is produced by heme oxygenase, the Fenton reaction could be very effective. In consequence, a lot of free radicals are formed under these conditions. Therefore, metal ion chelation may lead to oxidation stress and DNA relaxation or degradation. Moreover, it was shown herein that the porin P1 precursor fragment may be considered a DNA-cleaving agent. These findings suggest that bacteria are actively involved in colon cancer progression via a radical-mediated mechanism.

Studied peptides L^1^ and L^2^ derived from the outer membrane protein P1 precursor from *F. nucleatum* subsp. *nucleatum* bind Cu(II) ions tightly and form thermodynamically stable {4N,1O} complexes. Both experimental and theoretical results show that various coordination modes and structures can be observed in the solution. Several functional groups within the molecule are involved in metal binding. However, nitrogen donors play the leading role in metal binding. They form short and strong interactions with copper(II) ions. Imidazole rings are responsible for the greatest number of metal-ligand interactions. However, all typical histidine-cation and amide nitrogen-cation interactions are assisted by interactions between oxygen and metal ions. Metal-nitrogen interactions lie in the 1.8–2.2 Å range, while supporting interactions with oxygen have a wider range of 1.8–2.6 Å. Cu(II)-L^1^ and Cu(II)-L^2^ complexes also have a rich network of hydrogen bonds that stabilizes the complexes. Both ligands form short regular fragments of the backbones—alpha-helical for L^1^ and alpha-helical and 3-10 helical for Ac-FGEHEHGRD-NH_2_. Nevertheless, the HB network of the L^2^ complexes is significantly richer due to the presence of Arg residue, which is responsible for 50% of HBs or more.

Both complexes produce reactive oxygen species when accompanied by hydrogen peroxide or ascorbic acid. However, in the case of addition H_2_O_2_, the amount of ROS formed is significantly higher than observed for uncomplexed copper(II) ions. It should be stressed that in Fenton-like reactions, Cu(III) ions may be formed. This oxidation state is stabilized by the peptide backbone, involving deprotonated peptide bonds, hydrogen bonds and the surrounding of the metal ion. This leads to efficient hydroxyl radical generation. The situation is different in the case of the addition of negatively charged ascorbate. This molecule seems to be repelled by the carboxylate groups in the coordination sphere of metal ions. Therefore, ROS production is less pronounced for the complexes than free copper(II) ions. Both complexes exhibit DNA cleavage abilities. Surprisingly, 500 µM L^2^ causes overall DNA degradation, although this is not specific. Most probably, the histidine residues interact with DNA and the acid-base cleavage of the phosphate backbone occurs.

## Figures and Tables

**Table 1:** Thermodynamic and spectroscopic parameters for deprotonation and Cu(II) complex formation of fragments of porin protein P1 in aqueous solution. T = 25 °C, I = 0.1 mol dm^−3^ (KCl). Standard deviations are given in parentheses.

Species	Potentiometry		UV-Vis		CD		EPR	
	Log*β* ^a^	pKa ^b^	Λ	Ε	Λ	Δε	A_‖_	g_‖_
			[nm]	[M^−1^ cm^−1^]	[nm]	[M^−1^ cm^−1^]	[G]	
Ac-AKGHEHQLE-NH_2_ (L^1^)								
CuH_2_L^1^	21.01(2)	6.13	255 ^sh^	39,870	231	−2.59	143	2.335
			700	40	255	+0.83		
CuHL^1^	14.88(2)	6.56	-	-	-	-	167	2.300
CuL^1^	8.32(2)	7.43	255 ^sh^ 600	49,210 97	221 248 292 ^sh^ 337 505 645	−6.66 +6.06 +2.55 −1.24 +0.43 −0.19	185	2.264
CuH_-1_L^1^	0.89(1)	9.88	255 ^sh^ 540	62,300 170	221 249 292 ^sh^ 337 400 510 644	−6.75 +7.79 +2.93 −1.80 +0.18 +0.45 −0.29	190	2.197
CuH_-2_L^1^	−8.99(2)	-	255 ^sh^ 540	62,349 170	221 249 292 ^sh^ 337 400 510 644	−6.75 +7.79 +2.93 −1.80 +0.18 +0.45 −0.29	193	2.188
Ac-FGEHEHGRD-NH_2_ (L^2^)								
CuH_2_L^2^	21.47(1)	5.61	250 ^sh^ 707	25,200 38	238 250 ^sh^	−7.26 −2.34	141	2.338
CuHL^2^	15.86(2)	6.65	250 ^sh^ 610	48,400 98	237 250 ^sh^ 650	−9.23 −4.90 +0.56	165	2.295
CuL^2^	9.21(2)	7.15	250 ^sh^ 575	48,620 106	235 250 ^sh^ 325 635	−14.71 −4.90 −1.23 +0.63	180	2.215
CuH_-1_L^2^	2.06(3)	9.11	255 ^sh^ 540	61,450 114	230 260 290 340 590 640	−14.80 +4.80 +2.63 −2.43 +0.50 −0.21	190	2.199
CuH_-2_L^2^	−7.05(2)	10.39	260 ^sh^ 540	70,140 178	230 250 280 315 610 690	−14.80 +2.53 +2.54 −2.50 +1.50 −1.11	190	2.199
CuH_-3_L^2^	−17.44(4)	-	260 ^sh^ 540	70,140 178	230 250 280 315 610 690	−14.80 +2.53 +2.54 −2.50 +1.50 −1.11	198	2.193

Note: ^a^ Overall stability constants (*β*) expressed by the equation: *β*(CuH_n_L) = [CuH_n_L]/[Cu^2+^][L][H^+^]^n^); standard deviations on the last digit of stability constants are given in parentheses; charges omitted for the sake of simplicity; ^b^ acid dissociation constants (pKa) expressed as: pKa = log*β*(CuH_n_L) − log*β*(CuH_n-1_L); sh shoulder.

**Table 2:** Metal-ligand distances in angstroms for Ac-AKGHEHQLE-NH_2_ and Ac-FGEHEHGRD-NH_2_ complexes.

	CuH_2_L	CuHL	CuL	CuH_-1_L
Ac-AKGHEHQLE-NH_2_ (L^1^)				
H6 (N_1_)	1.862	1.941	1.992	1.994
H4 (N_2_)		1.919	1.888	2.013
E9 (amide N_3_)			2.074	2.011
L8 (amide N_4_)				2.353
E9 O_1_	1.777	2.043	2.554	2.033
E9 O_2_		2.047		
Ac-FGEHEHGRD-NH_2_ (L^2^)				
H4 (N_1_)	1.829	1.829	1.833	1.910
H6 (N_2_)		1.851	2.210	1.828
H4 (amide N_3_)			1.872	1.839
H6 (amide N_4_)				1.919
H6 (carbonyl O)		1.834	1.887	1.912
E3 O_1_	1.910		1.836	

**Table 3:** Hydrogen bonds of Cu(II)-L^1^ complexes.

Residue	H..PA [Å]	PD-H..PA [deg]	Fragment
CuH_2_L^1^			
Ac..H4	1.718	159.8	O..H*-N*
Ac..E5	1.905	161.9	O..H*-N*
E5..K2	2.100	150.3	O..H-N (alpha helix)
G3..E5	1.906	154.5	N-H..O*
CuHL^1^			
Q7..E9	1.904	170.2	N*-H*..O*
E5..K2	1.833	162.6	O..H-N (alpha helix)
K2..G3	1.913	155.2	O*..H-N
L8..E5	1.968	160.1	O..H-N (alpha helix)
CuL^1^			
E5..K2	1.885	162.2	O..H-N (alpha helix)
K2..G3	1.980	168.3	O*..H-N
CuH_-1_L^1^			
K2..G3	1.914	169.4	O*..H-N
K2..H4	1.850	154.6	O*..H-N

Note: * mark atoms from side chains.

**Table 4:** Hydrogen bonds of Cu(II)-L^2^ complexes.

Residue	H..PA [Å]	PD-H..PA [deg]	Fragment
CuH_2_L^2^			
D9..H6	1.984	151.7	O*..H*-N*
D9..NH_2_ (C-terminus)	1.866	166.4	O*..H-N
R8..R8	1.837	165.9	N*-H*..O
R8..E3	1.831	167.4	N*-H*..O
R8..Ac	1.884	151.9	N*-H*..O
E3..G7	1.930	166.9	O*..H-N
E3..R8	1.792	175.5	O*..H-N
CuHL^2^			
D9..NH_2_ (C-terminus)	1.832	153.8	O*..H-N
R8..R8	1.856	158.3	N*-H*..O
R8..Ac	1.891	150.6	N*-H*..O
R8..E3	2.267	165.6	N*-H*..O
H6..F1	1.936	162.9	N*-H*..O
H6..E5	1.873	158.3	N-H..O*
CuL^2^			
D9..NH_2_ (C-terminus)	1.828	153.7	O*..H-N
R8..D9	1.848	153.7	N*-H*..O
R8..H6	1.765	168.8	N*-H*..O
E5..R8	2.061	172.3	O..H-N (3-10 helix)
G7..E5	1.718	167.4	N-H..O*
F1..E3	1.851	1.665	O..H-N (3-10 helix)
CuH_-1_L^2^			
D9..NH_2_ (C-terminus)	1.813	111.2	O*..H-N
R8..D9	1.771	160.6	N*-H*..O
R8..E5	1.963	162.7	N*-H*..O
E5..G7	1.722	165.4	O*..H-N
E5..R8	1.901	162.3	O*..H-N
H4..E3	1.780	152.9	N-H..O*
F1..H4	1.803	159.5	O..H-N (alpha helix)

Note: * mark atoms from side chains.

## Data Availability

All data supporting the conclusions of this article are provided within and are available from the corresponding author.

## Supplementary Materials

The following are available online at https://www.mdpi.com/article/10.3390/ijms222212541/s1.
