# Supplementary material for: Effect of Copper(II) Ion Binding by Porin P1 Precursor Fragments from Fusobacterium nucleatum on DNA Degradation
Source: Int J Mol Sci. 2021 Nov 21;22(22):12541. doi: 10.3390/ijms222212541 (PMC8623562; doi:10.3390/ijms222212541)
Supplement: Supplementary file 1 [file ijms-22-12541-s001.zip › ijms-1449675-supplementary.pdf]

Supplementary Information

Table S1. Thermodynamic parameters for studied ligands deriving from porin protein P1 precursor, in aqueous solution. $T = 25$ °C, $I = 0.1$ mol dm^{-3} (KCl).

Ligand	logβ[a]	pKa[b]	deprotonating group
	**Ac-AKGHEHQLE–NH$_2$ (L^1)**		
H_5L	31.11(2)	4.06	-COOH (glutamic acid)
H_4L	27.05(3)	4.29	-COOH (glutamic acid)
H_3L	22.76(1)	6.18	$N_{imidazole}$ (histidine)
H_2L	16.58(2)	6.86	$N_{imidazole}$ (histidine)
HL	9.72(1)	9.72	$-NH_3^+$ (lysine)
	**Ac-FGEHEHGRD-NH$_2$ (L^2)**		
H_6L	34.10(1)	3.25	-COOH (aspartic acid)
H_5L	30.85(3)	3.73	-COOH (glutamic acid)
H_4L	27.12(1)	4.40	-COOH (glutamic acid)
H_3L	22.72(4)	6.16	$N_{imidazole}$ (histidine)
H_2L	16.56(1)	6.93	$N_{imidazole}$ (histidine)
HL	9.63(5)	9.63	guanidinium moiety $[-NH=C(NH_2)_2]^+$ (arginine)

[a] overall stability constants (β) expressed by the equation: $\beta(H_nL) = [H_nL] / [L][H^+]^n$); standard deviations on the last digit of stability constants are given in parentheses;
[b] acid dissociation constants (pK_a) expressed as: pK_a = $\log\beta\ (H_nL) - \log\beta\ (H_{n-1}L)$
